# Metabolite profiling and bioactivity guided fractionation of *Lactobacillaceae* and rice bran postbiotics for antimicrobial-resistant *Salmonella* Typhimurium growth suppression

**DOI:** 10.3389/fmicb.2024.1362266

**Published:** 2024-04-09

**Authors:** Nora Jean Nealon, Colette R. Worcester, Shea M. Boyer, Hannah B. Haberecht, Elizabeth P. Ryan

**Affiliations:** ^1^Department of Environmental and Radiological Health Sciences, College of Veterinary Medicine and Biomedical Sciences, Colorado State University, Fort Collins, CO, United States; ^2^Department of Veterinary Clinical Sciences, College of Veterinary Medicine, The Ohio State University, Columbus, OH, United States

**Keywords:** postbiotic, rice bran, metabolomics, Lactobacillaceae, bioactivity-guided fractionation, *Salmonella enterica* serovar Typhimurium, antimicrobial resistance

## Abstract

Probiotic-fermented supplements (postbiotics) are becoming increasingly explored for their activity against antibiotic-resistant enteropathogens. Prebiotics are often incorporated into postbiotics to enhance their efficacy, but due to strain differences in probiotic activity, postbiotic antimicrobial effects are poorly understood. To improve postbiotic antimicrobial efficacy, we investigated and compared metabolite profiles of postbiotics prepared with three lactic acid bacteria strains (*L. fermentum*, *L. paracasei*, and *L. rhamnosus*) cultured with and without rice bran, a globally abundant, rich source of prebiotics. At their minimum inhibitory dose, *L. fermentum* and *L. paracasei* postbiotics + rice bran suppressed *S*. Typhimurium growth 42–55% more versus their respective probiotic-alone postbiotics. The global, non-targeted metabolome of these postbiotics identified 109 metabolites increased in *L. fermentum* and *L. paracasei* rice bran postbiotics, including 49 amino acids, 20 lipids, and 12 phytochemicals metabolites. To identify key metabolite contributors to postbiotic antimicrobial activity, bioactivity-guided fractionation was applied to *L. fermentum* and *L. paracasei* rice bran-fermented postbiotics. Fractionation resulted in four *L. fermentum* and seven *L. paracasei* fractions capable of suppressing *S*. Typhimurium growth more effectively versus the negative control. These fractions were enriched in 15 metabolites that were significantly increased in the global metabolome of postbiotics prepared with rice bran versus postbiotic alone. These metabolites included imidazole propionate (enriched in *L. fermentum* + rice bran, 1.61-fold increase; *L. paracasei* + rice bran 1.28-fold increase), dihydroferulate (*L. fermentum* + rice bran, 5.18-fold increase), and linoleate (*L. fermentum* + rice bran, 1.82-fold increase; *L. paracasei* + rice bran, 3.19-fold increase), suggesting that they may be key metabolite drivers of *S*. Typhimurium growth suppression. Here, we show distinct mechanisms by which postbiotics prepared with lactic acid bacteria and rice bran produce metabolites with antimicrobial activity capable of suppressing *S*. Typhimurium growth. Probiotic strain differences contributing to postbiotic antimicrobial activity attract attention as adjunctive treatments against pathogens.

## 1 Introduction

*Salmonella enterica* serovar Typhimurium is a leading global cause of infectious diarrhea. Escalating levels of antimicrobial resistance across non-typhoidal *Salmonella* isolates complicates containment and treatment efforts, and consequently, alternative methods of microbial control are needed (CDC, 2019). A rapidly growing body of research examines probiotics as widespread gut health-promoting dietary supplements and native gut commensal microorganisms that may prevent and reduce *Salmonella* outbreaks (Luoma et al., 2017; Nealon et al., 2017a). More recently, probiotics have been combined with prebiotics, including purified carbohydrates and whole foods. Among whole food prebiotics, rice bran, milled from brown rice, is a phytochemically diverse source of lipids, amino acids, and vitamins/cofactors and contains prebiotic carbohydrates (Salmeron, 2017; Zarei et al., 2017; Nealon and Ryan, 2019). Dietary supplementation with rice bran was shown to enhance the growth of probiotic Lactobacillaceae in people, mice, pigs, and broiler chickens and reduce *Salmonella* shedding (Rubinelli et al., 2017; Sheflin et al., 2017; Zarei et al., 2017; Nealon et al., 2017a; Zambrana et al., 2019).

An emerging area of dietary supplement research includes postbiotics, which are the cell-free products of probiotics, including small molecules, cell wall components, microbial proteins, and extracellular polysaccharides (Cuevas-González et al., 2020). Postbiotics have been explored for their roles in immune modulation, as antioxidants, chemopreventive agents, and antimicrobial adjuncts and alternatives (Kumbhare et al., 2023). Specifically, postbiotics, prepared with lactic acid bacteria, a diverse group of probiotic strains native to human and animal microbiotas, have been increasingly explored for their roles in antimicrobial activity, including as anti-biofilm agents for interference with quorum sensing, for enhancing the growth of other beneficial microbial symbionts, as applications to food products or used, and in animal production systems to attenuate antimicrobial use (Ali et al., 2023; Chuah et al., 2023; Kudra et al., 2023; Kumbhare et al., 2023; Penchuk et al., 2023; Sepordeh et al., 2023; Sharafi et al., 2023; Tong et al., 2023). Across postbiotic studies, there remains a knowledge gap in understanding postbiotic mechanisms of action, including incomplete characterization of the bioactive small molecules driving these diverse functions (Kumbhare et al., 2023). This includes how both probiotic strain and prebiotic selection impact the function of postbiotics (Ali et al., 2023; Kumbhare et al., 2023).

Metabolomics, the systematic evaluation of small, bioactive molecules in living systems, is a tool that can improve our understanding of postbiotic functionality. When applied to microbial products and other biologics, metabolomics has expanded the suite of small molecules (metabolites) that we can detect and identify from microbial products and fermented foods (Shaffer et al., 2017). Specifically, a postbiotic prepared with probiotic *Lacticaseibacillus paracasei,* an established lactic acid probiotic strain, and rice bran enhanced *S*. Typhimurium growth suppression compared with an *L. paracasei* postbiotic alone, and this was associated with increases in 84 metabolites, predominantly lipid, amino acid, and phytochemical compounds, which had previously reported antimicrobial functions (Nealon et al., 2017a). In a second study, a postbiotic prepared with *Lacticaseibacillus rhamnosus* suppressed the growth of antimicrobial-resistant *S*. Typhimurium, *Escherichia coli*, and *Klebsiella oxytoca*, and *in silico* metabolic flux analysis of the postbiotic global metabolome revealed that amino acid metabolites were key contributors to its antimicrobial activity (Hove et al., 2023). Given that non-targeted metabolomics often identify numerous potential functional compounds, bioactivity-guided fractionation, which chromatographically subsets complex, functional, and natural substances into smaller suites of metabolites, can additionally be applied to postbiotics to subset and identify major contributors to their antimicrobial activity (Najmi et al., 2022). In these instances, fractionated postbiotics can be applied to a target of interest, such as *S*. Typhimurium, and modulations in *S*. Typhimurium growth with different postbiotic fractions can be identified and further profiled using metabolomics to identify the metabolite subsets in these fractions. These high-throughput and sensitive tools were applied herein to systematically characterize postbiotics that have functional bioactivity against antimicrobial-resistant *S*. Typhimurium.

The objective of this study was to functionally compare the bioactive small molecules present in postbiotics produced by three strains of Lactobacillaceae and rice bran. The overarching hypothesis of this study is that Lactobacillaceae + rice bran postbiotics produce distinct suites of small molecules that suppress multidrug-resistant *S*. Typhimurium growth. Targeted postbiotic preparations may become promising treatments and strategic preventive agents against antimicrobial-resistant pathogen outbreaks.

## 2 Materials and methods

### 2.1 Bacterial strains and culture preparation

Three Lactobacillaceae strains isolated from human fecal and colon tissue samples were purchased from ATCC (Manassas, VA): *Limosilactobacillus fermentum* ATCC 23271, *Lacticaseibacillus paracasei* ATCC 27092, and *Lacticaseibacillus rhamnosus* ATCC 21052. *Salmonella enterica* subsp. *enterica* serovar Typhimurium was provided by Dr. Sangeeta Rao from Colorado State University. The antimicrobial-resistance phenotype of this isolate is shown in Supplementary File S1 and was established using broth microdilution assay methods, which were standardized by the National Antimicrobial Resistance Monitoring System for Enteric Bacteria (CLSI, 2010). Before use, all bacteria were stored at −80°C as 1 mL aliquots with 20% glycerol (Avantor, Radnor, PA) with autoclaved de Man Rogosa Sharpe (MRS) broth (Becton, Dickinson and Company, Difco Laboratories, Franklin Lakes, NJ) for Lactobacillaceae strains or Luria–Bertani (LB) broth (MOBIO Laboratories Inc., Carlsbad, CA) for *S*. Typhimurium.

### 2.2 Rice bran extraction and media preparation

Rice bran extract was prepared using heat-stabilized Calrose rice bran (USDA-ARS Rice Research Unit, Stuttgart, AK), as previously described (Forster et al., 2013). Heat stabilization of rice bran was completed in a commercial dryer for 30 min at 110°C. In total, 4 g of rice bran was extracted in 42.6 mL of 80% aqueous solution of ice-cold (−80°C) methanol, vigorously vortexed for 5 min (232 Vortexer Fisher Scientific, Pittsburgh, PA, USA), incubated overnight at −80°C, and centrifuged at 4,000 *g* for 5 min (Beckman Coulter Allegra X-14R). The supernatant was collected and dried in a speedvac concentrator (SPD1010, Thermo Scientific, Pittsburgh, PA) at 45°C for approximately 48 h. To prepare MRS + rice bran extract, 100 μg of rice bran extract was added to 1 mL of MRS broth and autoclaved using a sterilization time of 45 min. Broth was stored at 4°C until use. The concentration of MRS + rice bran extract was the same as previous dose response studies of MRS + rice bran extract broth on *S*. Typhimurium growth (Nealon et al., 2017a).

### 2.3 Preparation of Lactobacillaceae postbiotics in the presence and absence of rice bran

To create postbiotics for all Lactobacillaceae and Lactobacillaceae + rice bran treatments, cell-free supernatant was prepared as described previously (Nealon et al., 2017a). In brief, 1×10^6^ CFU ml^−1^ (colony forming units) of *L. fermentum*, *L. paracasei*, or *L. rhamnosus* was grown for 24 h to mid/late logarithmic phase, added to 15 mL of MRS broth or 15 mL of MRS broth +100 μg ml^−1^ rice bran, and incubated at 37°C for 24 h. A, 100 μg ml^−1^ dose of rice bran extract was selected from previous studies, whereby 100 μg ml^−1^ of rice bran extract significantly suppressed *S*. Typhimurium growth compared with a rice bran extract-free control (Nealon et al., 2017a). Following the 24 h incubation, when all cultures were in the stationary growth phase, cultures underwent two rounds of centrifugation at 4,000 *g* for 10 min to separate the supernatant from the remaining bacterial pellet. The supernatant was adjusted to a pH of 4.5, which approximates the lower limit of acidity tolerated by *S*. Typhimurium (Chung and Goepfert, 1970), to control the effect of pH-dependent changes on *S*. Typhimurium growth. The supernatant was filter-sterilized through a 0.22-μm pore (Pall Corporation Life Sciences Acrodisc syringe filters, Port Washington, NY) and stored as 1 mL aliquots at −80°C until use. Supernatant sterility was confirmed prior to use by screening it for the absence of any growth at 37°C with repeated OD600 reads every 20 min for 24 h. A minimum of three replicates of each Lactobacillaceae and Lactobacillaceae + rice bran postbiotic were used to conduct each experiment described herein.

### 2.4 *Salmonella* Typhimurium growth suppression assay

The assay for *S*. Typhimurium growth suppression with Lactobacillaceae +/− rice bran postbiotics, including the selected dose range, was adapted from previously reported methods (Nealon et al., 2017a). *S*. Typhimurium was grown in LB broth at 37°C, until it reached early/mid logarithmic growth phase, as determined by repeated optical density reads at 600 nm. Lactobacillaceae and Lactobacillaceae + rice bran postbiotic supernatants were tested for dose-dependent growth suppression effects on *S*. Typhimurium. Approximately 2×10^6^ *S*. Typhimurium were added to sterile LB in a 96-well plate, and different concentrations of supernatant (12–25% per volume of LB) were added to each well. This range of concentrations was selected to identify a range of doses over which postbiotic supernatants exhibited no enhanced growth suppression of *S*. Typhimurium versus the vehicle controls (12%) to a dose, where all treatments showed an equivalent minimal growth of *S*. Typhimurium (25%) through the 16 h of the assay. The vehicle controls were either MRS (for Lactobacillaceae) or MRS + 100 μg ml^−1^ rice bran extract (for Lactobacillaceae + rice bran) that were added to LB broth. The negative control was *S*. Typhimurium inoculated into equivalent volumes of LB. All controls were adjusted to a pH of 4.5, which approximates the lower limit of acidity tolerated by *S*. Typhimurium (Chung and Goepfert, 1970).

To measure *S*. Typhimurium growth over time, OD600 was measured at 37°C every 20 min for 16 h. To quantify *S*. Typhimurium growth suppression, the percentage difference in growth suppression was calculated between pairs of treatments at 16 h using the following formula:

$$\frac{\left(OD600\;\mathrm{Treatment}\;1-OD600\;\mathrm{Treatment}\;2\right)\;}{\left(OD600\;\mathrm{Treatment}\;2\right)}*100\%$$

Each experiment contained a minimum of two technical replicates per treatment dose, and each experiment was repeated a minimum of three times.

### 2.5 Fractionation of Lactobacillaceae + rice bran cell-free supernatant

Given the increased efficacy of *L. fermentum* + rice bran and *L. paracasei* + rice bran postbiotics against *S*. Typhimurium versus their respective probiotic-alone postbiotics, each of these treatments underwent further chromatographic separation for elucidation of key metabolites driving their antimicrobial activity. In total, 5 ml of *L. fermentum* + rice bran and *L. paracasei* + rice bran postbiotics were each separated into 24 fractions using reverse-phase flash chromatography on a Combiflash^®^ RF+ Flash Chromatography Purification System (Teledyne ISCO, Thousand Oak, California). The 5-ml starting volume was determined following a range-finding analysis that optimized sample injection volume for metabolite recovery during non-targeted metabolomics analysis (data not shown). The stationary phase column was a C18-aq, 15.5 g-Gold Redisep column (Teledyne ISCO, Thousand Oaks, California), and the mobile phase gradient consisted of a water:methanol solution that increased in hydrophobicity over the course of the separation. To account for machine and batch variability in postbiotic antimicrobial activity, each supernatant was fractionated three times using a minimum of three batches collected on different days. UV absorbance detected at 214 and 254 nm wavelengths was compared across each fractionation run, to confirm consistency in chromatographs between biological and technical replicates. *L. fermentum* and *L. paracasei* cell-free supernatants were fractionated using the same column and run conditions. Following separation, fractions were dried at 55°C under a sterile fume hood and then re-constituted in 5 mL LB broth titrated to a pH of 4.5. All re-constituted fractions were stored at −80°C until use.

### 2.6 *Salmonella* Typhimurium growth suppression with postbiotic fractions

In total, 1×10^6^ CFU of each *S*. Typhimurium was added to LB broth on a 96-well plate and treated with each re-constituted fraction to create a 22% v/v concentration in LB, which was a Lactobacillaceae + rice bran postbiotic dose that previously exhibited growth suppression for this *S*. Typhimurium isolates (Nealon et al., 2017a). In each assay, *S*. Typhimurium inoculated in 4.5 pH-adjusted LB was used as the negative control. To adjust for starting differences in fraction optical densities and confirm media sterility over the course of the assay, blank LB and blank fraction + LB were included as controls. Each assay contained a minimum of two technical replicates for each treatment and was repeated three times for each *S*. Typhimurium isolate incubated with either *L. fermentum* + rice bran or *L. paracasei* + rice bran postbiotic supernatants.

### 2.7 Quantification and statistical analysis of *Salmonella* Typhimurium growth suppression assays with postbiotics

For analysis of unfractionated supernatants, a repeated-measures two-way analysis of variance was used to examine treatment and time-dependent differences in supernatant growth suppression when comparing Lactobacillaceae + rice bran with their respective Lactobacillaceae postbiotic treatments. For analysis of bioactive supernatant fractions, the OD600 of each fraction at each timepoint was compared with that of the negative control. In both analyses, significance was defined as *p* < 0.05 following *p*-value adjustment with a Tukey’s post-hoc test. All statistical analyses for these assays were performed using GraphPad Prism Version 10.1.1 (La Jolla, CA).

### 2.8 Postbiotic and postbiotic fraction metabolomics processing

The global, non-targeted metabolite profiles of each Lactobacillaceae supernatant, Lactobacillaceae + rice bran supernatant, vehicle control, and vehicle control + rice bran were performed by Metabolon Inc^©^ (Durham, NC). Selected fractions for postbiotic *L. fermentum* + rice bran (fractions 18, 21, and 22) and *L. paracasei* + rice bran (fractions 18, 21, 22, and 24) additionally underwent non-targeted metabolomics profiling. These fractions were selected for profiling because they exhibited differential magnitudes of *S*. Typhimurium growth suppression compared with the vehicle control treatment. In brief, all samples were shipped on dry ice to Metabolon and frozen at −80°C until sample processing with ultra-high-performance liquid chromatography tandem mass spectrometry (UPLC-MS/MS). Samples were re-solubilized in methanol, centrifuged at room temperature, and separated into five aliquots for downstream analysis: two aliquots for reverse-phase chromatography coupled with positive ion mode electrospray ionization (ESI), one aliquot for reverse phase chromatography coupled with negative ion mode ESI, a fourth aliquot for hydrophilic-interaction UPLC-MS/MS coupled with negative ion mode ESI, and the fifth aliquot saved as a back-up sample. Quality control samples were prepared by pooling similar aliquots across all fraction samples to account for chromatographic drift across subsequent UPLC-MS/MS runs.

Before injection, each aliquot was dried using an automated evaporation system (TurboVap^®^, LV Automated Evaporation System, Thermo Scientific, Pittsburgh, PA). Each dried sample was re-constituted, mixed with internal standard compounds of known concentration, and processed for the following UPLC-MS/MS workflows: Acidic positive ion mode conditions optimized for hydrophilic metabolite extraction with a C18 column (Waters UPLC BEH C18-2.1×100 mm, 1.7 μm) stationary phase and a mobile phase solution of water and methanol with 0.05% v/v perfluoropentanoic acid and 0.1% v/v formic acid; acidic positive ion mode conditions optimized for hydrophobic metabolite extraction with the same C18 column stationary phase as the previous condition and a mobile phase solution of methanol, acetonitrile, and water with 0.05% perfluoropentanoic acid and 0.01% formic acid; basic negative ion mode conditions with a C18 column (Waters UPLC BEH C18-2×100 mm, 1.7 μm) stationary phase and a mobile phase solution of methanol and water adjusted to a pH of 8 with ammonium bicarbonate; negative ESI coupled with a hydrophilic interaction stationary phase column (Waters UPLC BEH Amide 2.1×150 mm, 1.7 μm) and a mobile phase solution of water and acetonitrile adjusted to a pH of 10.8 with ammonium formate. All workflows used Waters AQUITY ultra-performance liquid chromatography columns coupled to a Thermo Scientific Q-Exactive high resolution mass spectrometers equipped with a heated ESI source and an Orbitrap mass analyzer set to a 35,000 mass:charge (m/z) resolution, with a tandem mass spectrometry setup that fluxed between dynamic exclusion MS and data-dependent MS^n^ scans covering 70–1000 m/z.

Raw mass spectral data were extracted using software developed by Metabolon where data were peak-extracted and normalized using area under the curve abundances with reference to quality control samples and internal standard recoveries. Mass spectral features were identified to known compounds using their retention indices, accurate masses (+/− 10 parts per million), and their MS/MS forward and reverse scores compared with Metabolon’s internal compound library containing ~3,300 purified chemical standards. Metabolite identities were cross-validated using the online mass spectral databases (Human Metabolome Database, “HMDB”; Kyoto Encyclopedia of Genes and Genomes, “KEGG,” and PubChem) (UniProt Consortium, 2018; Wishart et al., 2018; Kanehisa et al., 2019). For compounds that did not have a matching internal standard with the Metabolon library, identifies were directly made using these public databases.

### 2.9 Statistical analysis and data visualization of postbiotic and postbiotic fraction metabolomes

To examine the differences in metabolite abundance across Lactobacillaceae and Lactobacillaceae + rice bran postbiotics, raw metabolite abundances for each sample were median-scaled across the dataset and used for downstream statistical analysis. A Welch’s *t*-test with a Benjamini–Hochberg false discovery rate correction was used to identify metabolites that were differentially abundant between the following pairs of treatments: *L. fermentum* + rice bran versus *L. fermentum, L. paracasei +* rice bran versus *L. paracasei*, and *L. rhamnosus* + rice bran versus *L. rhamnosus.* Statistical significance was defined as *p* < 0.05 following false discovery rate (*q*-value) adjustment of *p*-values. Treatment fold differences were calculated by dividing the average median-scaled abundance of one treatment by the second treatment. Data visualization was performed using GraphPad Prism (version 10.1.1) and Metaboanalyst version 5.0 (Pang et al., 2021). To show treatment differences between the global, non-targeted metabolomes of postbiotics with and without rice bran, a partial least squares discriminant analysis (PLS-DA) plot was generated using median-scaled metabolite abundances across the top three components with a 5-fold cross-validation error rate. An integrated heat map and unsupervised hierarchical clustering analysis were generated to visualize the top 50 metabolites with the highest variable importance scores from the PLS-DA model. Hierarchical clustering analysis branch points were calculated using Euclidean distances and ward scaling. Supplementary File S2 provides the R script used for Metaboanalyst visualization.

For metabolite profiles of bioactive fractions, the metabolite raw abundances for each fraction were median-scaled across all *L. fermentum* samples or *L. paracasei* samples. Z-scores for each metabolite were calculated as previously described (Borresen et al., 2017; Zarei et al., 2017). In brief, Z-scores were obtained by subtracting the metabolite median-scaled abundance across all samples from the median-scaled abundance of each fraction, and this difference was then divided by the standard deviation of all *L. fermentum* or *L. paracasei* samples. Metabolites with a Z-score of ≥1.00 in each fraction were defined as enriched in these fractions relative to unfractionated supernatant. Data visualization for postbiotic fractionation metabolome data was completed using GraphPad Prism.

## 3 Results

### 3.1 Lactobacillaceae + rice bran postbiotics differentially suppress the growth of antimicrobial-resistant *Salmonella* Typhimurium

Lactobacillaceae and Lactobacillaceae + rice bran postbiotics exhibited concentration-dependent growth suppression on *S*. Typhimurium when applied over a concentration range of 12 to 25% v/v. Table 1 shows the relative percent efficacy of each Lactobacillaceae + rice bran versus Lactobacillaceae postbiotic for each of the three tested probiotic strains. Supplementary Table S3 shows growth suppression levels of each postbiotic versus the control treatment. There was a dose-dependent effect of postbiotics on *S*. Typhimurium growth suppression for all treatments (Lactobacillaceae alone or in combination with rice bran) compared with the vehicle controls and negative controls (*p* < 0.05) (Supplementary Table S4).

**Table 1:** Percent difference in antimicrobial-resistant *Salmonella* Typhimurium growth suppression when comparing Lactobacillaceae and Lactobacillaceae + rice bran postbiotics at different treatment concentrations.

	*S*. Typhimurium percent growth suppression			
Postbiotic supernatant concentration (% volume)	12%	18%	22%	25%
*L. fermentum* + Rice Bran / *L. fermentum*	**↑23.82% ± 7.71%**	**↑42.47% ± 10.93%**	30.04% ± 19.57%	18.21% ± 6.81%
*L. paracasei* + Rice Bran / *L. paracasei*	12.22% ± 1.87%	**↑55.21% ± 9.72%**	27.41 ± 9.24%	21.87 ± 2.29%
*L. rhamnosus* + Rice Bran / *L. rhamnosus*	13.94% ± 9.10%	5.76% ± 2.82%	29.15% ± 13.94%	**↓65.45% ± 72.95**

Postbiotic cell-free supernatant treatment concentrations were selected to span a range over which growth suppression was observable by Lactobacillaceae and/or Lactobacillaceae + rice bran treatments (12% volume of *S*. Typhimurium incubation media) versus the vehicle control treatments (de Man Rogosa Sharpe broth with and without rice bran extract) to which no difference between treatments could be readily observed (25% volume of *S*. Typhimurium incubation media). Growth suppression values for each supernatant dose represent the average value for a minimum of three independent experiments ± standard deviation measured at 16 h post-incubation (end point for the assay). Bold treatments indicate statistically significant (*p* < 0.05) growth suppression differences between treatments at 16 h following two-way analysis of variance with Tukey’s *post-hoc* test. ↑ indicates treatment in the numerator exhibited increased *S*. Typhimurium growth suppression compared with the treatment in the denominator; ↓ indicates treatment in the numerator exhibited decreased *S*. Typhimurium growth suppression compared with the treatment in the denominator.

**Figure 1:**
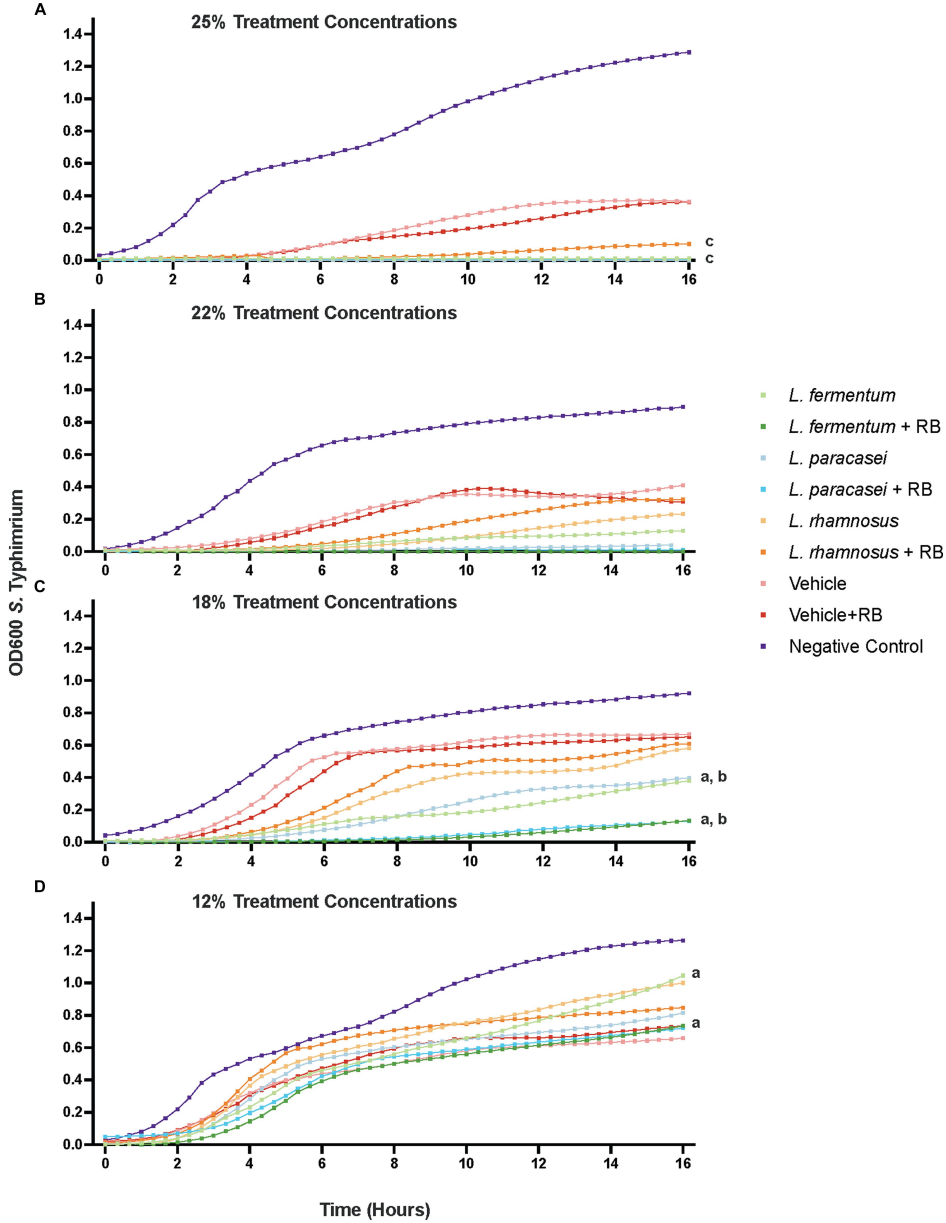
Probiotic and probiotic *+* rice bran postbiotics differentially suppress the growth of antimicrobial-resistant *S*. Typhimurium in a dose-dependent manner. Growth suppression by postbiotic supernatants are depicted at a 25% v/v concentration **(A)**, 22% concentration **(B)**, 18% concentration **(C)**, and 12% concentration **(D)**. Optical density (OD600) at each time point represents the mean of at least three independent experiments with a minimum of three technical replicates per experiment. Letters denote treatments that are significantly different (*p* < 0.05) for 16 h following a two-way repeated measures analysis of variance with a Tukey’s post-hoc correction: a. *L. fermentum* + rice bran versus *L. fermentum*; b. *L. paracasei* + rice bran versus *L. paracasei*; c. *L. rhamnosus* + rice bran versus *L. rhamnosus*. RB, Rice Bran.

The results are presented in Figure 1 for the 18% supernatant concentration, as it was the lowest concentration at which any postbiotic produced significantly enhanced 16 h growth suppression compared with the vehicle controls. At 16 h, the 18% *L. paracasei* + rice bran postbiotic was 55.21% more effective at suppressing *S*. Typhimurium growth compared with the *L. paracasei*-only postbiotic (*p* < 0.0001). *L. fermentum* + rice bran significantly suppressed *S*. Typhimurium growth by 42.47% more than the *L. fermentum*-only postbiotic (*p* < 0.0001). At all tested doses, the *L. rhamnosus* + rice bran postbiotic did not enhance *S*. Typhimurium growth suppression at any point during the assay when compared with the *L. rhamnosus* postbiotic.

### 3.2 Rice bran differentially modulates the metabolism of Lactobacillaceae strains

**Figure 2:**
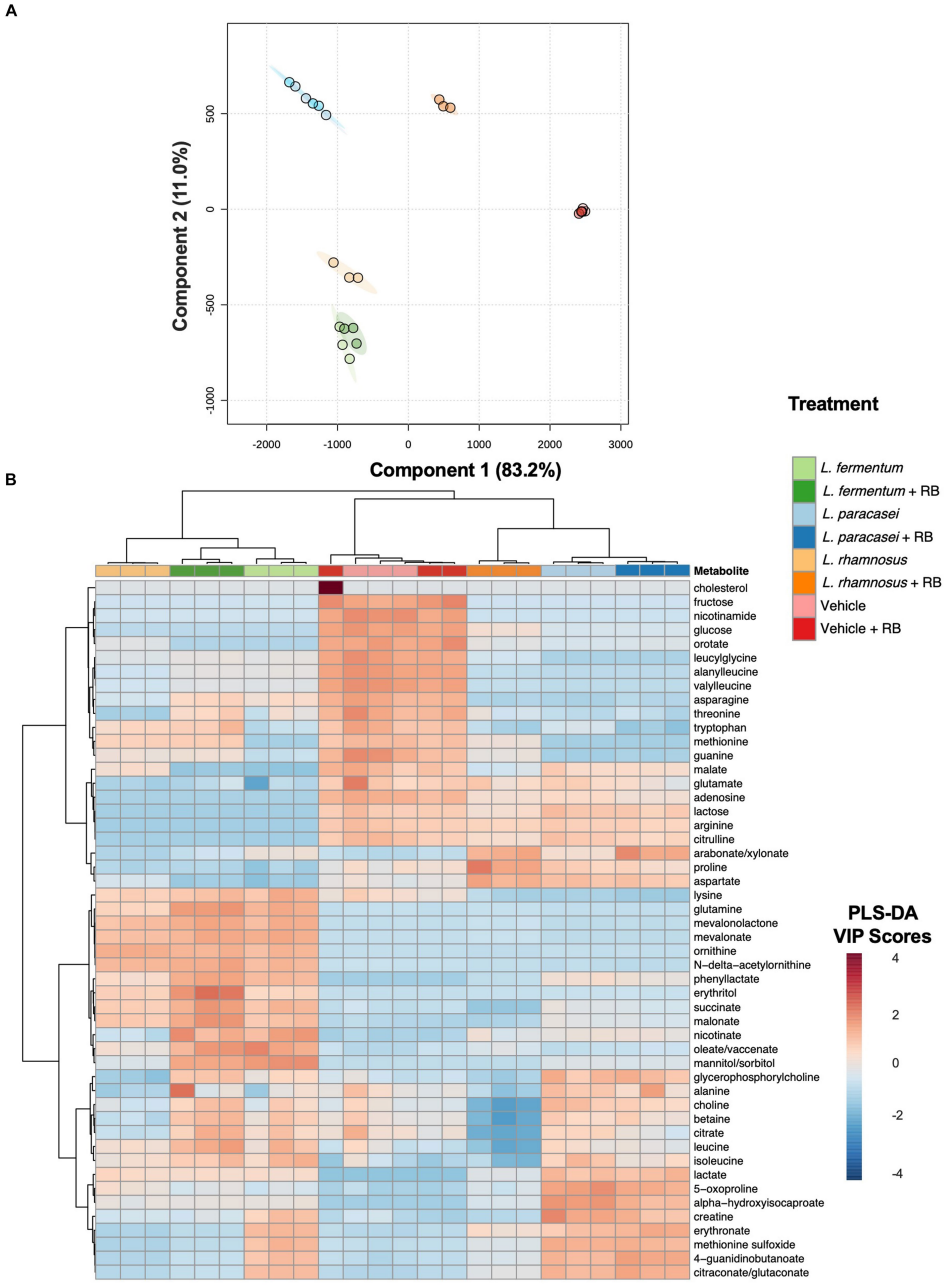
Probiotic metabolomes are differentially modulated during rice bran fermentation. Partial least squares discriminant analysis (PLS-DA) projection for components 1 and 2. Each circle represents an individual sample, and ellipses indicate the 95% confidence interval around each treatment group **(A)**. Heat map + unsupervised hierarchical clustering analysis of postbiotic treatments prepared with and without rice bran and vehicle controls (de Man Rogosa Sharpe broth with and without rice bran extract) **(B)**. Metabolites with the 50 largest variable importance (VIP) scores from the partial least squares discriminant analysis model are visualized. Red cells indicate a higher average VIP score for the metabolite in the respective sample when compared with other samples. Blue cells indicate a lower average VIP score for the metabolite when compared with other samples. Branch points are calculated using Euclidean distances and are shown with Ward scaling. All visualizations use median-scaled metabolites.

Global non-targeted metabolomics identified 381 metabolites in Lactobacillaceae and Lactobacillaceae + rice bran postbiotics, including 325 metabolites that were differentially abundant when comparing each postbiotic prepared with rice bran with its probiotic-only treatment (Supplementary File S4). Differentially abundant metabolites included 122 amino acids, 32 peptides, 29 carbohydrates, 9 energy metabolites, 42 lipids, 48 nucleotides, 27 phytochemical/other, and 17 vitamins/cofactors. PLS-DA analysis (Figure 2A) indicated that the largest variation in metabolite profiles, with separation along Component 1 (83.2% of the variation), occurred primarily between postbiotic treatments versus the vehicle control and secondarily between postbiotic strains relative to each other. Component 2 (11.0% of the variation) primarily separated Lactobacillaceae from Lactobacillaceae + rice bran postbiotics for each treatment group. The *L. rhamnosus* versus *L. rhamnosus* + rice bran postbiotic treatment groups exhibited the largest distance from each other along both components. To identify metabolites contributing to treatment differences, unsupervised hierarchical clustering analysis compared Lactobacillaceae + rice bran and Lactobacillaceae postbiotics, where the 50 metabolites with the highest PLS-DA VIP scores across treatments are shown in Figure 2B. Clear separation between postbiotic treatments prepared with different postbiotic strains were identified, including when comparing each postbiotic with its postbiotic + rice bran treatment. Amino acid metabolites were the most highly represented across postbiotics and accounted for ~48% of these visualized metabolites. Lipids (~18%) and carbohydrates (~14%) were the second and third most abundant chemical classes represented. Other metabolite classes contributing to postbiotic differences included energy metabolites (~12%), vitamins/cofactors (~4%), and nucleotides (~4%).

Given the enhanced antimicrobial activity of *L. paracasei* + rice bran and *L. fermentum* + rice bran postbiotics against *S*. Typhimurium, there was particular interest in postbiotic metabolites increased during rice bran fermentation. Supplementary File S5 shows metabolites that were significantly increased in the global, non-targeted metabolome of *L. fermentum* + rice bran and *L. paracasei* + rice bran postbiotics relative to their probiotic-only postbiotic treatments. For the *L. fermentum* + rice bran postbiotic, 148 metabolites were significantly increased relative to the *L. fermentum* postbiotic, including the carbohydrate glucose (7.75-fold increase, *p* = 0.0074), fatty acids azelate (1.24-fold increase, *p* = 0.0220), and linoleate (1.82-fold increase, *p* = 0.0084) and the rice bran phytochemical dihydroferulic acid (5.18-fold increase, *p* = 0.0024). Multiple methionine metabolites exhibited large increases in *L. fermentum* + rice bran versus *L. fermentum* postbiotics including methionine (89.76-fold increase, *p* = 8.94E-05), N-formylmethionine (15.06-fold increase, *p* = 0.0005), and N-acetylmethionine (9.72-fold increase, *p* = 0.0010). In the *L. paracasei* + rice bran postbiotic, 32 metabolites were significantly increased relative to the *L. paracasei* postbiotic. These metabolites included histidine metabolite imidazole propionate (1.28-fold increase, *p* = 0.0400), carbohydrate sucrose (7.39-fold increase, *p* = 0.0001), fatty acid linoleate (3.19-fold increase, *p* = 0.0482), and rice bran phytochemical 4-hydroxybenzoate (1.41-fold increase, *p* = 0.0147).

### 3.3 Postbiotic fractions prepared from *Lacticaseibacillus paracasei +* rice bran and *Limosilactobacillus fermentum* + rice bran exhibited growth suppressive activity against *Salmonella* Typhimurium

**Figure 3:**
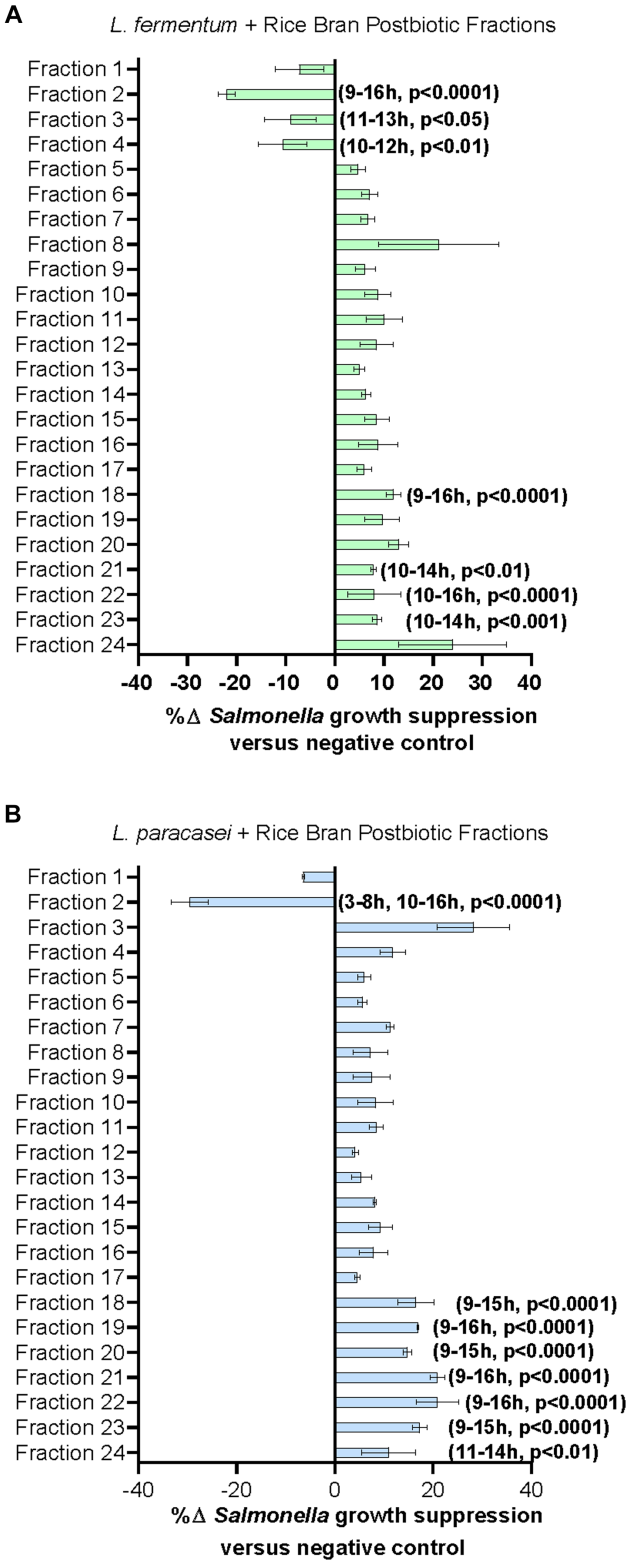
Lactobacillaceae+ rice bran supernatant postbiotic fractions suppress *S*. Typhimurium growth. **(A)** *L. fermentum* + rice bran postbiotic and **(B)**: *L. paracasei* + rice bran postbiotic fraction metabolite profiles and fraction percent *S*. Typhimurium growth suppression. Percent growth suppression of fractions is relative to the negative control treatment (Luria–Bertani broth). Values reflect maximal percent difference achieved by the fraction over the time interval where it was significantly different in growth compared with the negative control. Time intervals are rounded to the nearest hour. Parentheses indicate the time interval when the fraction exhibited significantly different growth, where positive values indicate increased *S*. Typhimurium growth suppression relative to the negative control, and negative values reflect decreased *S*. Typhimurium growth suppression relative to the negative control.

Given the large number of metabolites that could potentially be contributing to the enhanced antimicrobial activity of the *L. fermentum* + rice bran and *L. paracasei* + rice bran postbiotics relative to their probiotic-alone postbiotics, these postbiotics underwent fractionation to subset metabolites. Each of the 24 fractions created from these postbiotics were subsequently applied to *S*. Typhimurium and screened for growth suppression activity. Figure 3 shows the maximal percent growth suppression achieved for each *L. fermentum* + rice bran postbiotic fraction (Figure 3A) and *L. paracasei* + rice bran postbiotic fraction (Figure 3B). Table 2 summarizes the maximal percent differences for all fractions that suppressed *S*. Typhimurium compared with the negative control. Four fractions of the *L. fermentum* + rice bran postbiotic exhibited *S*. Typhimurium growth suppression: fraction 18 (11.39% more effective versus negative control, *p* < 0.0001), fraction 21 (7.83%, *p* < 0.005), fraction 22 (12.79%, *p* < 0.0001), and fraction 23 (8.01%, *p* < 0.001). Seven *L. paracasei* + rice bran postbiotic fractions suppressed *S*. Typhimurium growth: fraction 18 (15.95% more effective, *p* < 0.0001), fraction 19 (16.74%, *p* < 0.0001), fraction 20 (17.70%, *p* < 0.0001), fraction 21 (22.30%, *p* < 0.0001), fraction 22 (19.90%, *p* < 0.0001), fraction 23 (17.05%, *p* < 0.0001), and fraction 24 (10.96%, *p* < 0.01). Collectively, *L. fermentum* + rice bran and *L. paracasei* + rice bran postbiotic fractions with growth inhibition activity achieved maximal *S*. Typhimurium suppression between 10 and 12 h post-incubation, approximately during the late exponential growth phase of *S*. Typhimurium.

**Table 2:** Growth suppression of antimicrobial-resistant *Salmonella* Typhimurium by postbiotic supernatant fractions compared with the negative control.

Bioactive fraction	Mobile phase solvent		*S*. Typhimurium percent growth suppression	
	% Water	% Methanol	*L. fermentum* + rice bran postbiotic	*L. paracasei* + rice bran postbiotic
18	12.5%	87.5%	**↑ 11.39% ± 2.82%*(12 h)***	**↑ 15.95% ± 6.99%*(12 h)***
19	0%	100%	9.11% ± 5.32%*(4 h)*	**↑ 16.74% ± 0.80%*(12 h)***
20	0%	100%	8.70% ± 10.52%*(2 h)*	**↑ 17.70% ± 5.45%*(12 h)***
21	0%	100%	**↑ 7.83% ± 0.97%***(12 h)*	**↑ 22.30% ± 3.88%*(12 h)***
22	0%	100%	**↑ 12.79% ± 8.68%*(12 h)***	**↑ 19.90% ± 5.62%*(12 h)***
23	100%	0%	**↑ 8.01% ± 4.83%*(10 h)***	**↑ 17.05% ± 2.08%*(10 h)***
24	100%	0%	23.28% ± 19.92%*(12 h)*	**↑ 10.96% ± 9.53%*(12 h)***

Percent differences show maximum percent difference in *S*. Typhimurium growth for fractions versus the negative control (Luria–Bertani broth). Times in parentheses reflect the hour at which the fraction achieved maximal growth difference compared with the negative control, rounded to the nearest hour. The results reflect three independent experiments for each *S*. Typhimurium isolate with each treatment mean ± standard deviation. Treatments are bolded when growth was significantly different (*p* < 0.05), following a two-way analysis of variance test with Tukey’s post-hoc correction. ↑ indicates that fraction had significantly more *S*. Typhimurium growth suppression compared with the negative control. Fraction numbers not shown did not have significantly increased *S*. Typhimurium growth suppression relative to the negative control for either the *L. fermentum* + rice bran or *L. paracasei* + rice bran postbiotics.

### 3.4 Metabolite composition for the bioactive postbiotic *Lactobacillaceae* + rice bran fractions

Non-targeted metabolite profiling was completed on selected postbiotic fractions exhibiting *S*. Typhimurium growth suppression in both *L. fermentum +* rice bran (fractions 18, 21, and 22) and *L. paracasei* + rice bran (fractions 18, 21, 22, and 24) and was selected to represent different portions of the mobile phase extraction gradient (different % water versus % methanol solvent ratio). A total of 196 distinct metabolites were identified in the *L. fermentum* + rice bran postbiotic fractions, including 182 metabolites in fraction 18, 138 metabolites in fraction 21, and 123 metabolites in fraction 22. In the *L. paracasei* + rice bran postbiotic fractions, 222 total metabolites were identified, including 167 metabolites in fraction 18, 158 metabolites in fraction 21, 162 metabolites in fraction 22, and 137 metabolites in fraction 24.

**Figure 4:**
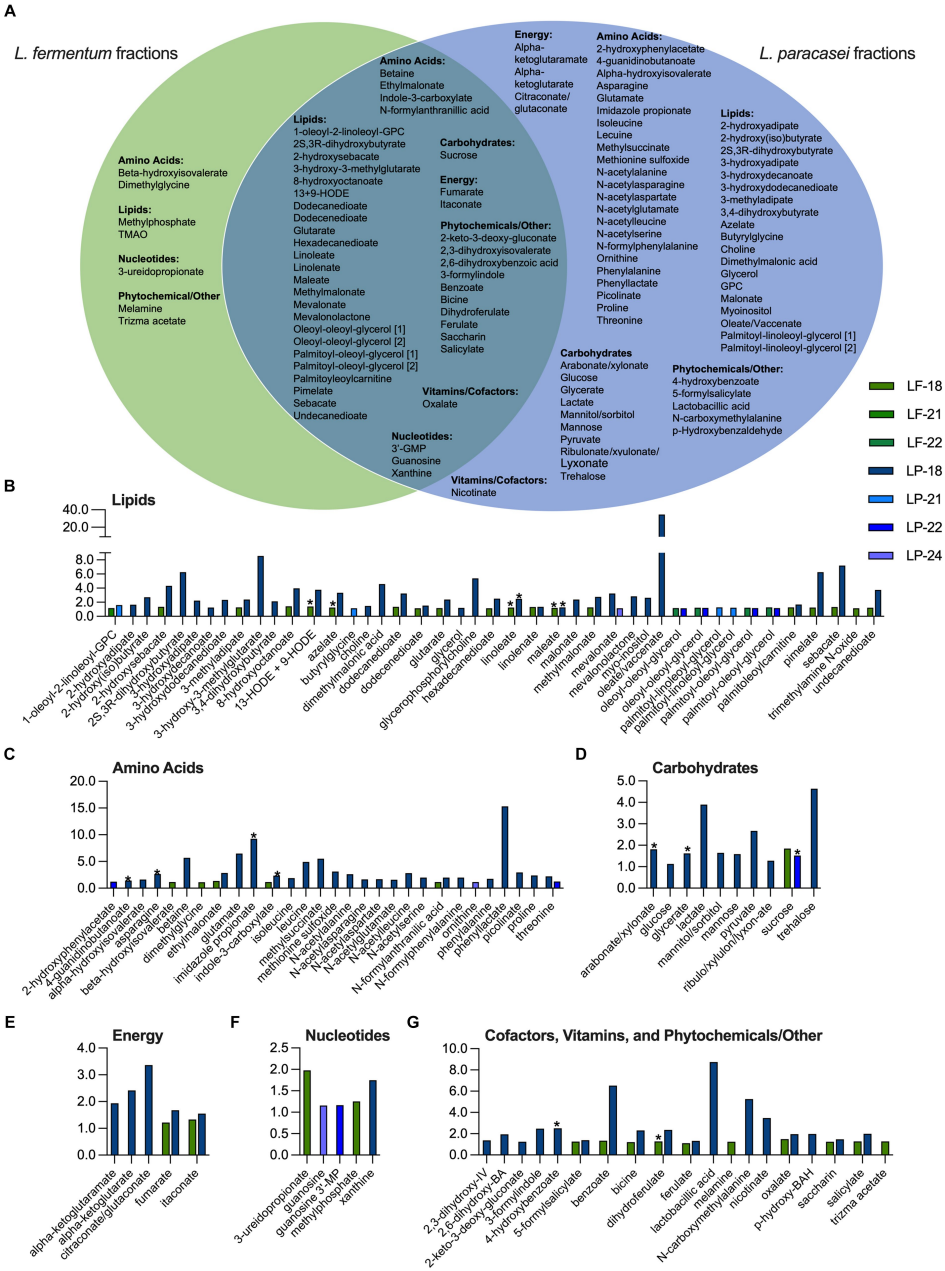
*L. fermentum +* rice bran and *L. paracasei +* rice bran postbiotics produce *S*. Typhimurium growth-suppressive fractions with distinct profiles of enriched metabolites. **(A)** Metabolites that were enriched Z-score ≥ 1.00 in *S*. Typhimurium-suppressive fractions for *L. fermentum* + rice bran (fractions 18, 21, and 22) and the *L. paracasei* + rice bran (fractions 18, 21, 22, and 24) postbiotics that were distinct to each postbiotic versus enriched in both postbiotic treatments. **(B-G)** Metabolite Z-scores for enriched metabolites in the *L. fermentum* + rice bran (green bars) and *L. paracasei* + rice bran (blue bars) postbiotics. * indicates metabolite that was significantly increased in the global, non-targeted metabolome of *L. fermentum* + rice bran versus *L. fermentum* and/or *L. paracasei* + rice bran versus *L. paracasei*. BA, benzoate/benzoic acid; BAH, benzaldehyde; GMP, guanosine monophosphate; IV, isovalerate; LF, *L. fermentum*; LP, *L. paracasei*; MP, monophosphate; GPC, glycerophosphorylcholine; HODE, hydroxyoctadecadienoic acid; RB, rice bran; TMAO, trimethylamine N-oxide; [1] and [2] identify metabolites with isomers.

To identify metabolites that had increased the abundance in postbiotic fractions relative to unfractionated postbiotics, the metabolite abundances in *S*. Typhimurium suppressing fractions were compared with the complete (un-fractionated) metabolomes of *L. fermentum* + rice bran and *L. paracasei* + rice bran postbiotics. A metabolite was defined as enriched its relative abundance in a fraction had a Z-score ≥ 1.0 when compared with the metabolite abundance in the respective unfractionated postbiotic. Figure 4 and these metabolites enriched in *L. fermentum* + rice bran and *L. paracasei* + rice bran postbiotic fractions and these are additionally detailed with Z-scores and by fraction in Supplementary File S6. *L. fermentum +* rice bran and *L. paracasei* + rice bran postbiotic bioactive fractions were enriched in 43 and 106 total metabolites, respectively (Figure 4A). For *L. fermentum* + rice bran, this included 38 metabolites in fraction 18, 1 metabolite in fraction 21, and 4 metabolites in fraction 22. For *L. paracasei* + rice bran, this included 91 metabolites in fraction 18, 4 metabolites in fraction 21, 8 metabolites in fraction 22, and 3 metabolites in fraction 24. For both postbiotics, lipids contributed the largest number enriched metabolites (~48% of metabolites in *L. fermentum* + rice bran and ~ 40% in *L. paracasei* + rice bran). A total of 2 enriched lipid metabolites were distinct to *L. fermentum* + rice bran, 19 lipid metabolites were distinct to *L. paracasei* + rice bran, and 24 lipid metabolites were shared by both postbiotics. These lipids included the fatty acids, such as azelate (Z-score of 1.24 in *L. fermentum* + rice bran fraction 18 and 3.34 in *L. paracasei* + rice bran fraction 18), oleate/vaccenate (Z-score of 34.52 in *L. paracasei* + rice bran fraction 18), and linoleate (Z-score of 1.24 in *L. fermentum* + rice bran fraction 18, 2.47 in *L. paracasei* + rice bran fraction 18). In addition, it is noteworthy that the mevalonate lipid, 3-hydroxy-3-methylglutarate, was enriched in *L. paracasei* + rice bran fraction 18 (Z-score of 8.54) (Figure 4B).

Among other enriched metabolites, amino acids contributed to 12% of the *L. fermentum* + rice bran-enriched metabolites, ~34% of *L. paracasei* + rice bran-enriched metabolites, phytochemicals to ~21% of *L. fermentum* + rice bran, and ~ 14% to *L. paracasei* + rice bran-enriched metabolites (Figure 4 and Supplementary File S6). Enriched amino acid metabolites included the histidine metabolite imidazole propionate (Z-score of 9.23 in *L. paracasei* + rice bran fraction 18), the glycine metabolite dimethylglycine (Z-score of 1.11 in *L. fermentum* + rice bran fraction 18), and the amino acid threonine (Z-score of 2.21 in *L. paracasei* + rice bran fraction 18, 1.25 in *L. paracasei* + rice bran fraction 22). Enriched phytochemical metabolites included the rice bran-derived dihydroferulate (Z-score 1.27 in *L. fermentum* + rice bran fraction 18, 2.35 in *L. paracasei* + rice bran fraction 18) and salicylate (Z-score of 1.27 in *L. fermentum* + rice bran fraction 18, 1.98 in *L. paracasei* + rice bran fraction 18).

## 4 Discussion

The overarching goal of this study was to examine the antimicrobial activity of three postbiotic preparations prepared with and without rice bran, a prebiotic source, on antimicrobial-resistant *S*. Typhimurium, which is the leading global cause of diarrhea in people and animals (CDC, 2019). This study identified two postbiotic preparations of Lactobacillaceae + rice bran that showed enhanced growth suppression of antimicrobial-resistant *S*. Typhimurium versus their respective probiotic-only postbiotic treatments (Figure 1 and Table 1). Among these treatments, postbiotics produced by *L. fermentum* + rice bran and *L. paracasei* + rice bran demonstrated ~42–55% enhanced *S*. Typhimurium growth suppression compared with their respective Lactobacillaceae postbiotics at a minimum dose of 18% supernatant/v (Table 1 and Figure 1).

Although *L. rhamnosus* + rice bran and *L. rhamnosus* postbiotics were both effective at suppressing *S*. Typhimurium growth relative to their vehicle controls (Supplementary File S2), the *L. rhamnosus* + rice bran postbiotic did not exhibit enhanced *S*. Typhimurium growth suppression versus *L. rhamnosus* postbiotic at any tested dose (Figure 1 and Table 1), highlighting that prebiotics differentially act on probiotic strains to modulate their functionality. While rice bran did not enhance the antimicrobial activity of the *L. rhamnosus* postbiotic against *S*. Typhimurium, it may have enhanced *L. rhamnosus* functions in other ways not captured in this study, as evidenced by the considerable non-targeted metabolome changes observed when comparing the *L. rhamnosus* and the *L. rhamnosus* + rice bran postbiotic metabolite profiles (Figure 2). These mechanisms include the impacts on pathogen colonization and interactions with the immune system, which have been demonstrated in previous studies using combinations of *L. rhamnosus* and rice bran (Yang et al., 2015; Lei et al., 2016; Nealon et al., 2017b).

A role for metabolites in the enhanced antimicrobial activity of *L. fermentum* + rice bran and *L. paracasei* + rice bran postbiotics was supported by the 109 metabolites that were significantly increased when comparing these postbiotics with their respective Lactobacillaceae-only treatments (Figure 2 and Supplementary Files S4, S5). The large number of differentially abundant amino acid and lipid metabolite compounds is consistent with previous studies, which concluded that these chemical classes contributed to the largest metabolomic changes during microbial fermentation of rice bran (Sheflin et al., 2015; Nealon et al., 2017a,b, 2019; Demissie et al., 2020; Seyoum et al., 2021). The substantial enhancement of methionine metabolites in the *L. fermentum* + rice bran postbiotic relative to the *L. fermentum* postbiotic has not been previously explored for its roles in antimicrobial activity. However, strain-dependent uptake and metabolism of environmental methionine have been reported for *L. fermentum* strains (Hossain, 2022), where various lactic acid bacteria strains utilize methionine and its derivatives to produce antimicrobial peptides and/or convert methionine into various downstream fatty acids or other metabolites with potential bioactivity (Rubinelli et al., 2017; Bindu and Lakshmidevi, 2021; Heredia-Castro et al., 2021; Hossain, 2022; Meng et al., 2022). Given these similarities across studies and the large number of differentially abundant metabolites potentially contributing to their antimicrobial activity, these two postbiotics were chromatographically fractionated and further evaluated for key metabolites, driving their antimicrobial activity against *S*. Typhimurium.

Bioactivity-guided fractionation identified four *L. fermentum* + rice bran and seven *L. paracasei* + rice bran postbiotic fractions that exhibited between ~8 and 22% enhanced *S*. Typhimurium growth suppression relative to the negative control (Figure 3 and Table 2), suggesting that they share some of the antimicrobial features of the un-fractionated postbiotic + rice bran supernatants. To investigate this further, the metabolite profiles of selected postbiotic fractions were examined and contained 43 enriched metabolites in *L. fermentum* postbiotic fractions and 106 metabolites in *L. paracasei* + rice bran postbiotic fractions (Figure 4 and Supplementary File S6). Similar to the global metabolite profile of these postbiotics, lipid, amino acid, and phytochemical metabolites comprised the majority of these enriched metabolites, including those metabolites that were dually significantly increased in the *L. fermentum* + rice bran and *L. paracasei* + rice bran postbiotics versus their respective probiotic-only postbiotic. Specifically, significant increases in the fatty acids, including azelate (*L. fermentum* + rice bran postbiotic) and linoleate (both *L. fermentum* + rice bran and *L. paracasei* + rice bran postbiotics), amino acids (methionine, *L. fermentum* + rice bran postbiotic), the phytochemicals ferulate and salicylate (enriched in both *L. fermentum* + rice bran and *L. paracasei* + rice bran postbiotics) have been demonstrated to increase during lactic acid bacteria metabolism of rice bran (Nealon et al., 2017a,b, 2019; Demissie et al., 2020; Seyoum et al., 2021) and are associated with enhanced antimicrobial and antiviral activity of these postbiotics when applied to *S*. Typhimurium and other enteropathogens (Rubinelli et al., 2017; Nealon et al., 2017a,b). The dual increase in these metabolites in the non-targeted global metabolome and enrichment in bioactive fraction metabolomes of postbiotics herein support the central roles of these metabolites and chemical classes in driving probiotic + rice bran postbiotic antimicrobial activity.

Limitations of this investigation include incomplete characterization and comparison of volatile organic compounds and short chain fatty acids such as lactate, acetate, propionate, and butyrate, as well as antimicrobial peptides, which are all known contributors to the antimicrobial activity of Lactobacillaceae (Pithva et al., 2011; Ibrahim et al., 2021). These chemical classes were not explored because the UPLC-MS/MS approach used herein was not optimized to capture these metabolites. Future experiments can include fermentation systems that better capture volatile organic compounds, before they partition out of each postbiotic, and couple these with gas chromatography approaches (Meredith and Tfaily, 2022). Proteomics approaches can additionally be applied to characterize antimicrobial peptides derived from probiotics and postbiotics, and they can be integrated with metabolic network analysis and metaboloics analysis to understand the relationships between the proteome and metabolome in conferring antimicrobial activity (Hove et al., 2023). Ultimately, incorporating these metabolite classes will allow for more robust characterization of postbiotic antimicrobial activity, including examination how their production changes with the addition of rice bran to each postbiotic preparation.

Another limitation includes the examination of only a subset of all bioactive fractions with demonstrated antimicrobial activity against *S*. Typhimurium. It is possible that with metabolomic characterization of additional bioactive fractions, a more comprehensive understanding of postbiotic strain differences will develop. For example, additional bioactive fractions may contain enriched levels of methionine metabolites in the *L. fermentum* + rice bran postbiotic that was significantly increased in the global, non-targeted metabolome but not identified among enriched metabolites in *L. fermentum* + bioactive fractions profiled herein. With a more thorough understanding of the enriched bioactive metabolites in these other fractions, future studies can consequently apply targeted metabolomics to identify the concentrations of these compounds to use in downstream testing as purified cocktails of postbiotic compounds for targeted antimicrobial therapy. While no postbiotic fractions were capable of producing *S*. Typhimurium growth suppression levels similar to un-fractionated postbiotic + rice bran supernatants, it is additionally possible that combinations of bioactive fractions and/or their enriched metabolites will provide antimicrobial synergy that mirror the unfractionated postbiotic preparations if screened together in future assays. Furthermore, comparisons between postbiotics with and without rice bran in this study were limited to metabolites with known identities. While characterized metabolites provided robust numbers of treatment differences herein, it is widely reported that many metabolites in non-targeted datasets, including microbial metabolites, are still uncharacterized (Bauermeister et al., 2022; Zhou et al., 2022). In addition to the known metabolites identified herein, future evaluations using these methods may identify different and/or additional antimicrobial metabolites in postbiotics, including explaining strain differences in postbiotic antimicrobial activity. It should be noted that adjustment of all postbiotic treatments to a pH of 4.5, to control the impact of pH on *S*. Typhimurium growth, may have impacted the bioactivity of each postbiotic, such that the antimicrobial activity of any metabolite could be pH-dependent. For example, the metabolites salicylate and acetate have been shown to differentially impact the susceptibility of *E. coli* to the antibiotic kanamycin depending on the culture pH, and the bioactivity of probiotic-derived antimicrobial peptides has been shown to be pH-dependent when applied to various pathogens (Aumercier et al., 1990; Amiri et al., 2022). Future experiments could examine antimicrobial metabolites and peptides over different pH ranges to better characterize their full spectrum of *S*. Typhimurium growth suppressive activity.

The antimicrobial metabolites identified in preparations of Lactobacillaceae + rice bran postbiotics have targeted functional potential for use in industrial, environmental, medical, and veterinary settings. Given the rise in antimicrobial resistance pressures in the environment, alternative and novel sources of safe antimicrobials are urgently needed in people, animals, and agricultural systems. The systematic examination of postbiotics to prevent and treat the spread of antimicrobial-resistant infections is of current and emerging importance to global health. Postbiotic production using probiotics that are native to mammals, alongside widely available prebiotics from foods such as rice bran, is a promising area for future application to the problem of antimicrobial resistance. The application of both non-targeted metabolomics and bioactivity-guided fractionation to well-studied probiotic strains and rice bran has filled knowledge gaps of postbiotic metabolic diversity and provides rationale for developing predictive models that assess postbiotic metabolic capacity. Increasing our understanding of postbiotic metabolite production can fuel the development of targeted microbial-based preventives and treatments against multidrug-resistant pathogens that include but are not limited to *S*. Typhimurium.

## Data availability statement

The raw data supporting the conclusions of this article will be made available by the authors, without undue reservation.

## Author contributions

NN: Conceptualization, Data curation, Formal analysis, Funding acquisition, Investigation, Methodology, Supervision, Validation, Visualization, Writing – original draft, Writing – review & editing. CW: Conceptualization, Data curation, Formal analysis, Investigation, Methodology, Validation, Writing – original draft, Writing – review & editing. SB: Data curation, Formal analysis, Investigation, Validation, Writing – original draft, Writing – review & editing. HH: Data curation, Formal analysis, Investigation, Validation, Writing – original draft, Writing – review & editing. ER: Conceptualization, Data curation, Formal analysis, Funding acquisition, Investigation, Methodology, Project administration, Resources, Supervision, Validation, Visualization, Writing – original draft, Writing – review & editing.
