# Supplementary material for: Metabolite profiling and bioactivity guided fractionation of Lactobacillaceae and rice bran postbiotics for antimicrobial-resistant Salmonella Typhimurium growth suppression
Source: Front Microbiol. 2024 Apr 9;15:1362266. doi: 10.3389/fmicb.2024.1362266 (PMC11040457; doi:10.3389/fmicb.2024.1362266)
Supplement: Supplementary file 1 [file Data_Sheet_1_1.docx]

| **File S1.** *Salmonella* Typhimurium antimicrobial resistance profile | | |
| --- | --- | --- |
| **Antimicrobial Agent** | **Susceptibility** | **Minimum Inhibitory  Concentration  (µg mL^-1^)** |
| Amoxicillin-Clavulanate | Resistant | >32 |
| Ampicillin | Resistant | >32 |
| Azithromycin | Susceptible | 2 |
| Ceftiofur | Resistant | >8 |
| Chloramphenicol | Resistant | >32 |
| Ciprofloxacin | Susceptible | 0.5 |
| Sulfisoxazole | Resistant | >256 |
| Tetracycline | Resistant | >32 |
| Trimethoprim-sulfamethoxazole | Resistant | >4 |
| Minimum inhibitory concentrations for each agent reflect the lowest antimicrobial concentration at which visual microbial growth was prevented. Susceptibility designations of “Susceptible” and “Resistant” were based on Clinical Laboratory Standards Institute guidelines for Enterobacteriaceae. Abbreviations: µg mL^-1^ = micrograms per milter | | |
