# Supplementary material for: Metabolite profiling and bioactivity guided fractionation of Lactobacillaceae and rice bran postbiotics for antimicrobial-resistant Salmonella Typhimurium growth suppression
Source: Front Microbiol. 2024 Apr 9;15:1362266. doi: 10.3389/fmicb.2024.1362266 (PMC11040457; doi:10.3389/fmicb.2024.1362266)
Supplement: Supplementary file 2 [file Data_Sheet_2_1.docx]

# PID of current job: 1715427

mSet<-InitDataObjects("specbin", "stat", FALSE)

mSet<-Read.TextData(mSet, "Replacing_with_your_file_path", "colu", "disc");

mSet<-SanityCheckData(mSet)

mSet<-ReplaceMin(mSet);

mSet<-SanityCheckData(mSet)

mSet<-FilterVariable(mSet, "F", 25, "none", -1)

mSet<-PreparePrenormData(mSet)

mSet<-Normalization(mSet, "MedianNorm", "NULL", "NULL", ratio=FALSE, ratioNum=20)

mSet<-PlotNormSummary(mSet, "norm_0_", "png", 72, width=NA)

mSet<-PlotSampleNormSummary(mSet, "snorm_0_", "png", 72, width=NA)

mSet<-PlotPCA2DScore(mSet, "pca_score2d_0_", "png", 72, width=NA, 1,2,0.95,0,0)

mSet<-PlotPCALoading(mSet, "pca_loading_0_", "png", 72, width=NA, 1,2);

mSet<-RF.Anal(mSet, 500,7,1)

mSet<-PlotRF.Classify(mSet, "rf_cls_0_", "png", 72, width=NA)

mSet<-PlotRF.VIP(mSet, "rf_imp_0_", "png", 72, width=NA)

mSet<-PlotRF.Outlier(mSet, "rf_outlier_0_", "png", 72, width=NA)

mSet<-PlotHeatMap(mSet, "heatmap_0_", "png", 72, width=NA, "norm", "row", "euclidean", "ward.D","bwm", 8, "overview", T, T, NULL, T, F, T, T, T)

mSet<-PlotSubHeatMap(mSet, "heatmap_1_", "png", 72, width=NA, "norm", "row", "euclidean", "ward.D","bwm", 8, "vip", 50, "overview", T, T, T, F, T, T, T)

mSet<-GetGroupNames(mSet, "null")

colVec<-c("#bbde94","#549d3e","#aecde1","#NA","#f3c17c","#ee8732","#ee9e9b","#d1382b")

shapeVec<-c(0,0,0,0,0,0,0,0)

mSet<-UpdateGraphSettings(mSet, colVec, shapeVec)

mSet<-PlotSubHeatMap(mSet, "heatmap_2_", "pdf", 72, width=NA, "norm", "row", "euclidean", "ward.D","bwm", 8, "vip", 50, "overview", T, T, T, F, T, T, T)

mSet<-PlotPLSPairSummary(mSet, "pls_pair_0_", "png", 72, width=NA, 5)

mSet<-PlotPLS2DScore(mSet, "pls_score2d_0_", "png", 72, width=NA, 1,2,0.95,0,0)

mSet<-PlotPLS3DScoreImg(mSet, "pls_score3d_0_", "png", 72, width=NA, 1,2,3, 40)

mSet<-PlotPLSLoading(mSet, "pls_loading_0_", "png", 72, width=NA, 1, 2);

mSet<-PlotPLS3DLoading(mSet, "pls_loading3d_0_", "json", 1,2,3)

mSet<-PlotPLS.Imp(mSet, "pls_imp_0_", "png", 72, width=NA, "vip", "Comp. 1", 15,FALSE)

mSet<-PLSDA.CV(mSet, "loo", 5,3, "Q2")

mSet<-PlotPLS.Classification(mSet, "pls_cv_1_", "png", 72, width=NA)

mSet<-SaveTransformedData(mSet)
