# Supplementary material for: Metabolite profiling and bioactivity guided fractionation of Lactobacillaceae and rice bran postbiotics for antimicrobial-resistant Salmonella Typhimurium growth suppression
Source: Front Microbiol. 2024 Apr 9;15:1362266. doi: 10.3389/fmicb.2024.1362266 (PMC11040457; doi:10.3389/fmicb.2024.1362266)
Supplement: Supplementary file 3 [file Data_Sheet_3_1.docx]

| **File S3.** Probiotic + Rice Bran and Probiotic postbiotic cell-free supernatant *Salmonella* Typhimurium percent growth suppression compared to vehicle control and negative control treatments | | | | |
| --- | --- | --- | --- | --- |
| **Supernatant Concentration (v/v)** | **25%** | **22%** | **18%** | **12%** |
| Vehicle Control /  Negative Control | ↑63.02% ±  0.64% | ↑58.56% ± 2.98% | ↑45.24% ± 12.89% | ↑48.60% ± 8.83% |
| Vehicle Control + Rice Bran /  Vehicle Control | ↓26.90% ± 10.31% | ↓140.58% ± 77.73% | ↑27.04% ± 9.38% | ↓26.74% ± 12.46% |
| Vehicle Control + Rice Bran / Negative Control | ↑59.25% ± 4.28% | ↑58.40% ± 7.23% | ↑18.87% ± 8.82% | ↑34.66% ± 5.76% |
| *L. fermentum*/  Vehicle Control | ↑76.66% ± 0.79% | ↑43.10% ± 19.17% | ↑67.47% ± 8.84% | ↑93.91% ± 29.26% |
| *L. fermentum* / Negative Control | ↑91.18% ± 0.059% | ↑75.71% ± 10.43% | ↑50.72% ± 10.47% | ↑51.80% ± 24.27% |
| *L. fermentum* + Rice Bran /  Vehicle Control + Rice Bran | ↑76.01% ± 1.56% | ↑84.64% ± 5.76% | ↑68.37% ± 7.53% | ↑8.12% ± 3.87% |
| *L. fermentum* + Rice Bran /  Negative Control | ↑89.72% ± 1.34% | ↑84.74% ± 0.63% | ↑72.09% ± 5.99% | ↑39.14%  0.060% |
| *L. paracasei* /  Vehicle Control | ↑67.72% ± 6.35% | ↑49.09% ± 10.22% | ↑66.84% ± 27.97% | ↑44.33% ± 14.43% |
| *L. paracasei* /  Negative Control | ↑90.69% ± 0.44% | ↑81.59% ± 2.01% | ↑57.18% ± 10.91% | ↑32.86% ± 5.88% |
| *L. paracasei* + Rice Bran /  Vehicle Control + Rice Bran | ↑59.83% ± 11.57% | ↑80.65% ± 5.62% | ↑71.60% ± 4.60% | ↑15.27% ± 6.23% |
| *L. paracasei* + Rice Bran /  Negative Control | ↑88.65% ± 0.64% | ↑84.63% ± 1.42% | ↑74.89% ± 3.62% | ↑34.90% ± 6.33% |
| *L. rhamnosus* /  Vehicle Control | ↑74.72% ± 0.90% | ↑35.24% ± 15.91% | ↑51.94% ± 1.85% | ↑68.73% ± 31.65% |
| *L. rhamnosus* /  Negative Control | ↑90.50% ± 0.79% | ↑63.22% ± 3.13% | ↑26.73% ± 6.56% | ↑20.87% ± 9.30% |
| *L. rhamnosus* + Rice Bran /  Vehicle Control + Rice Bran | ↑64.29% ± 8.35% | ↑70.14% ± 0.94% | ↑14.59% ± 2.15% | ↑20.96%± 4.51% |
| *L. rhamnosus* + Rice Bran /  Negative Control | ↑83.64% ± 5.48% | ↑53.83% ± 1.37% | ↑23.57% ± 5.64% | ↑32.04%± 1.30% |

*S.* Typhimurium growth (measured in optical density) was statistically evaluated over time and between treatments using a repeated-measure two-way analysis of variance with a Tukey posthoc adjustment. Significance was defined as p <0.05 following posthoc adjustment. Treatment differences are represented as a percent difference in *S.* Typhimurium growth suppression when comparing each pair of indicated treatments. Vehicle control treatments included de Man Rogosa Sharpe broth with and without rice bran extract. The negative control treatment was Luria Bertani broth. ↑ indicates treatment in the numerator exhibited increased growth suppression compared to the treatment in the denominator; ↓ indicates treatment in the numerator exhibited decreased growth suppression compared to the treatment in the denominator. Growth suppression values for each supernatant dose represent a minimum of three independent experiments with at least two technical replicates per experiment and are reported as mean ± standard error of the mean. All growth suppression values represent the level of growth suppression at 16h (assay endpoint). Abbreviations: v/v = percent by volume.
