# Supplementary material for: Metabolite profiling and bioactivity guided fractionation of Lactobacillaceae and rice bran postbiotics for antimicrobial-resistant Salmonella Typhimurium growth suppression
Source: Front Microbiol. 2024 Apr 9;15:1362266. doi: 10.3389/fmicb.2024.1362266 (PMC11040457; doi:10.3389/fmicb.2024.1362266)
Supplement: Supplementary file 5 [file Data_Sheet_5_1.docx]

| **File S5. Metabolites Increased in *L. fermentum* and *L. paracasei* postbiotics prepared with rice bran** | | | | |
| --- | --- | --- | --- | --- |
| **Chemical Class** | **Metabolic Pathway** | **Metabolite** | **Postbiotic Treatment Fold Differences** | |
|  |  |  | **LF + Rice Bran / LF** | **LP + Rice Bran / LP** |
| Amino Acid | Glycine, Serine, Threonine | Glycine | **1.04 *(0.0333)*** | 1.02 *(0.3762)* |
|  |  | Betaine | **1.04** ***(0.0266)*** | 1.04 *(0.1747)* |
|  |  | Serine | 1.10 ***(0.0066)*** | 1.03 *(0.2776)* |
|  |  | N-acetylserine | **1.22 *(0.0191)*** | 0.88 *(0.1026)* |
|  |  | Threonine | **1.09** ***(0.0277)*** | 1.07 *(0.1380)* |
|  |  | N-acetylthreonine | **1.20** ***(0.0112)*** | 1.07 *(0.3557)* |
|  | Alanine, Aspartate | Asparagine | **1.07 *(0.0163)*** | **1.17 *(0.0216)*** |
|  |  | N-acetylaspartate | **1.33 *(0.0238)*** | 1.06 *(0.4235)* |
|  |  | 3-Sulfo-L-alanine | **0.62 *(0.0084)*** | **2.35 *(0.0021)*** |
|  | Glutamate | Glutamine | **1.16 *(0.0005)*** | 1.12 *(0.1406)* |
|  |  | N-acetylglutamate | **1.28 *(0.0191)*** | 1.02 *(0.4172)* |
|  | Histidine | Histidine | **1.19 *(0.0070)*** | 0.90 *(0.1380)* |
|  |  | N-acetylhistidine | **0.87 *(0.0123)*** | 1.14 *(0.0744)* |
|  |  | Imidazole propionate | **1.61 *(0.0094)*** | **1.28** ***(0.0400)*** |
|  |  | Imidazole lactate | **1.66 *(0.0029)*** | 1.08 *(0.2583)* |
|  |  | Histamine | **1.09 *(0.0123)*** | 0.95 *(0.1079)* |
|  |  | 1-Methylhistamime | **1.28 *(0.0127)*** | 0.97 *(0.4357)* |
|  | Lysine | N2-acetyllysine | **1.59 *(0.0104)*** | 1.03 *(0.4975)* |
|  | Phenylalanine, Tyrosine | Phenylpyruvate | **1.90 *(0.00080)*** | 1.15 *(0.2820)* |
|  |  | Phenyllactate | **1.14 *(0.0052)*** | 0.93 *(0.2601)* |
|  |  | 4-Hydroxy-phenylpyruvate | **1.78 *(0.0238)*** | 1.07 *(0.4399)* |
|  | Tryptophan | Tryptophan | **1.23 *(0.0018)*** | 0.91 *(0.1246)* |
|  |  | Indoleacetate | **1.47 *(0.0119)*** | **0.69 *(0.0355)*** |
|  |  | Indole-3-carboxylic acid | **0.12 *(0.0036)*** | **1.94 *(0.0147)*** |
|  |  | Thioproline | **6.78 *(0.0008)*** | 0.82 *(0.1183)* |
|  | Leucine, Isoleucine, Valine | Leucine | **1.04 *(0.0193)*** | 1.04 *(0.1070)* |
|  | Methionine, Cysteine, S-adenosyl methionine, Taurine | Methionine | **89.76 *(8.94E-05)*** | 0.64 *(0.0809)* |
|  |  | N-acetylmethionine | **9.72 *(0.0010)*** | 0.97 *(0.3729)* |
|  |  | N-formylmethionine | **15.06 *(0.0005)*** | 1.02 *(0.4648)* |
|  |  | Methionine sulfone | **0.37 *(0.0033)*** | **8.30 *(0.0003)*** |
|  |  | S-methylcysteine | **1.28 *(0.0142)*** | 0.76 *(0.1178)* |
|  |  | Cysteine sulfinic acid | **3.47 *(0.0021)*** | 0.96 *(0.4975)* |
|  |  | Hypotaurine | **6.12 *(0.0008)*** | **0.46 *(0.0347)*** |
|  | Urea Cycle, Arginine and Proline | Arginine | **2.33 *(0.0162)*** | 1.01 *(0.4752)* |
|  |  | N-delta-acetylornithine | **1.11 *(0.0179)*** | 0.66 *(0.2672)* |
|  |  | N-alpha-acetylornithine | **1.55 *(0.0026)*** | 1.01 *(0.5106)* |
|  |  | 2-oxoarginine* | **3.51 *(0.0088)*** | 1.00 *(0.5539)* |
|  | Creatine | creatinine | **1.26 *(0.0076)*** | 1.08 *(0.1026)* |
|  | Polyamine | 4-acetamidobutanoate | **1.34 *(0.0036)*** | 1.10 *(0.2360)* |
|  |  | Putrescine | **1.01 *(0.0475)*** | 1.07 *(0.2359)* |
|  | Guanidino, Acetamido | 1-methylguanidine | **0.27 *(0.0023)*** | **1.35 *(0.0464)*** |
|  |  | 4-guanidinobutanoate | **0.39 *(0.0012)*** | **1.19 *(0.0286)*** |
|  | Gamma-glutamyl-Amino Acid | Gamma-glutamylglutamine | **1.17 *(0.0236)*** | 1.16 *(0.1365)* |
|  |  | Gamma-glutamylmethionine | **4.90 *(0.0008)*** | **-** |
|  | Dipeptide | Lysylleucine | **1.10 *(0.0342)*** | 0.95 *(0.5348)* |
|  |  | Threonylphenylalanine | **1.07 *(0.0132)*** | **-** |
|  |  | Tryptophylglycine | **1.79 *(0.0044)*** | **-** |
| Carbohydrate | Glycolysis, Gluconeogenesis, Pyruvate | Glucose | **7.75 *(0.0074)*** | **1.08 *(0.1365)*** |
|  |  | 3-phosphoglycerate | **1.43 *(0.0142)*** | 0.98 *(0.35)* |
|  |  | Pyruvate | **2.44 *(0.0009)*** | **1.01 *(0.4651)*** |
|  |  | Glycerate | **0.41 *(0.0097)*** | **1.07 *(0.0286)*** |
|  | Pentose | Ribonate | **0.67 *(0.0183)*** | **1.87 *(0.0216)*** |
|  |  | Arabonate/Xylonate | **0.51 *(0.0066)*** | **1.88 *(0.0450)*** |
|  | Disaccharide, Oligosaccharide | Sucrose | **-** | **7.39 *(0.0001)*** |
|  |  | Melibiose | **2.16 *(0.0016)*** | **-** |
|  | Aminosugar | N-acetylneuraminate | **1.56 *(0.0096)*** | 0.83 *(0.2326)* |
|  | Advanced Glycation End Product | Erythrulose | **5.46 *(1.44E-05)*** | 1.47 *(0.0734)* |
| Energy | Tricarboxylic Acid Cycle | Alpha-ketoglutarate | **2.49 *(0.0321)*** | 0.77 *(0.0919)* |
|  |  | Malate | **1.56 *(0.0333)*** | 0.99 *(0.5457)_* |
|  |  | Succinate | **1.08 *(0.0399)*** | 0.99 *(0.5119)* |
|  |  | Tricarballylate | 1.26 *(0.0563)* | **1.80 *(0.0031)*** |
|  | Long Chain  Fatty Acid | Eicosenoate | **1.29 *(0.0296)*** | 1.65 *(0.0221)* |
|  | Polyunsaturated Fatty Acid  (n3 and n6) | Linoleate | **1.82 *(0.0084)*** | 3.19 *(0.0482)* |
|  | Fatty Acid, Dicarboxylate | 2-hydroxyglutarate | **1.20 *(0.0236)*** | 1.04 *(0.3557)* |
|  |  | Maleate | **1.13 *(0.0193)*** | 1.45 *(0.0705)* |
|  |  | Azelate | **1.24 *(0.0220)*** | 1.19 *(0.0809)* |
|  | Fatty Acid Synthesis | Malonate | **1.20 *(0.0068)*** | 0.97 *(0.3634)* |
|  | Fatty Acid Metabolism, Acyl Carnitine | Acetylcarnitine | **1.35 *(0.0036)*** | 0.91 *(0.1070)* |
|  | Fatty Acid, Monohydroxy | 3-hydroxydecanoate | **1.20 *(0.0205)*** | 0.71 *(0.0502)* |
|  |  | 13+9 HODE | **4.20 *(0.0074)*** | 2.11 *(0.0502)* |
| Phospholipid | Choline | Choline | **1.05 *(0.0319)*** | 1.03 *(0.1291)* |
|  | Choline Phosphate | Choline Phosphate | **1.06 *(0.0342)*** | 1.02 *(0.4049)* |
|  | Glycerophosphoryl Choline | Glycerophosphoryl Choline | **1.06 *(0.0292)*** | 1.07 *(0.1819)* |
| Glycerolipid | Glycerolipid | Glycerol | **4.18 *(0.0008)*** | 1.08 *(0.3263)* |
|  |  | Glycerol-3-phosphate | **1.19 *(0.0068)*** | 1.06 *(0.0919)* |
| Nucleotide | (Hypo)Xanthine, Inosine | Urate | **1.76 *(0.0076)*** | - |
|  | Adenosine | Adenosine 5’-Monophosphate | **1.43 *(0.0021)*** | 0.99 *(0.5360)* |
|  |  | Adenine | **1.12 *(0.0342)*** | 1.16 *(0.1678)* |
|  |  | 2’-deoxyadenosine | **1.57 *(0.0334)*** | **1.31 *(0.0216)*** |
|  |  | N6-succinyladenosine | 0.95 *(0.1925)* | **1.51 *(0.0327)*** |
|  | Guanine | Guanine | **1.62 *(0.0005)*** | **1.16 *(0.0286)*** |
|  |  | Guanosine-2’,3’-cyclic monophosphate | **1.14 *(0.0386)*** | **1.11 *(0.1115)*** |
|  | Orotate | Orotidine | **1.62 *(0.0238)*** | - |
|  | Cytidine | Cytosine | **2.58 *(0.0119)*** | 0.98 *(0.3151)* |
|  |  | 2’-Deoxycytidine | **-** | **1.60 *(0.0331)*** |
| Vitamin/Cofactor | Nicotinate, Nicotinamide | Nicotinate ribonucleoside | **2.64 *(0.0183)*** | 1.18 *(0.2491)* |
|  |  | Nicotinamide | **1.14 *(0.0164)*** | 0.80 *(0.2820)* |
|  |  | Nicotinamide adenine dinucleotide | **2.98 *(0.0154)*** | 0.73 *(0.3557)* |
|  |  | Trigonelline | **1.12 *(0.0154)*** | 1.10 *(0.1183)* |
|  | Riboflavin | Riboflavin | **1.71 *(0.0083)*** | - |
|  |  | Flavin mononucleotide | **1.78 *(0.031)*** | 0.85 *(0.3557)* |
|  | Biotin | Biotin | **3.33 *(0.0319)*** | 0.65 *(0.0912)* |
|  | Vitamin B6 | Pyridoxamine | **1.89 *(0.0161)*** | 0.81 *(0.0842)* |
|  |  | Pyridoxal | **1.81 *(0.0238)*** | - |
| Phytochemical/Other | Benzoate | 4-hydroxybenzoate | **0.54 *(0.0083)*** | **1.41 *(0.0147)*** |
|  |  | p-Aminobenzoate | **1.54 *(0.0164)*** | 0.92 *(0.3550)* |
|  | Food Component/ Plant | Beta-guanidino-propanoate | **0.47 *(0.0005)*** | **1.74 *(0.0123)*** |
|  |  | Dihydroferulic acid | **5.18 *(0.0024)*** | 0.93 *(0.3557)* |
|  |  | Erythritol | **2.17 *(0.0036)*** | 1.20 *(0.1493)* |
|  |  | Histidinol | **1.51 *(0.0049)*** | 0.97 *(0.2897)* |
|  |  | Stachydrine | **1.13 *(0.0142)*** | 1.11 *(0.1724)* |
|  |  | Harmane | **0.36 *(0.0008)*** | **1.62 *(0.0031)*** |
|  |  | Pyrraline | **1.44 *(0.0024)*** | **1.24 *(0.045)*** |
|  |  | 2-keto-3-deoxy-gluconate | **4.51 *(0.0036)*** | 1.05 *(0.4975)* |
|  |  | Succinimide | **1.34 *(0.0217)*** | **1.23 *(0.0482)*** |
| Each box shows the metabolite fold difference for each postbiotic prepared with rice bran relative to its respective postbiotic-only supernatant. Fold differences were calculated by dividing the average median scaled abundance of the numerator treatment by that of the denominator. Numbers in italics are p-values, where significant metabolites are bolded. Significance was defined as p < 0.05 following false discovery rate calculations made on multiple Wilcoxon tests. Abbreviations: HODE =Hydroxyoctadecadienoic acid. LF = *L. fermentum*; LP = *L. paracasei.* * Indicates metabolite identity was made using *in-silico* mass spectral predictions. [brackets] indicate chemical isomers. | | | | |
